# Inhibition of Ubiquitin-Specific Proteases as a Novel Anticancer Therapeutic Strategy

**DOI:** 10.3389/fphar.2018.01080

**Published:** 2018-09-27

**Authors:** Tao Yuan, Fangjie Yan, Meidan Ying, Ji Cao, Qiaojun He, Hong Zhu, Bo Yang

**Affiliations:** Zhejiang Province Key Laboratory of Anti-cancer Drug Research, Institute of Pharmacology and Toxicology, College of Pharmaceutical sciences, Zhejiang University, Hangzhou, China

**Keywords:** ubiquitin-specific proteases, ubiquitin proteasome system, deubiquitinases, anticancer, inhibitors

## Abstract

Dysfunction or dysregulation of the ubiquitin proteasome system (UPS) is closely related to tumorigenesis and the development of multiple cancers. Targeting the UPS provides a new anticancer therapeutic strategy, but clinically available UPS-targeted inhibitors, including lenalidomide and bortezomib, are limited to treat solid tumors. Under physiological conditions, deubiquitinases or deubiquitinating enzymes (DUBs) play vital roles in the UPS by removing ubiquitin from substrate proteins and regulating their proteasomal degradation and sub-localization, thus maintaining the balance between ubiquitination and deubiquitination for protein quality control and homeostasis. The aberrant expression or function of DUBs generally leads to the occurrence and progression of a series of disorders, including malignant tumors. Therefore, targeting DUBs is a novel anticancer therapeutic strategy. Ubiquitin-specific proteases (USPs) are the largest subfamily of DUBs which have attracted considerable interest as anticancer targets. Most of USPs are abnormally activated or expressed in a variety of malignant tumors or in the tumor microenvironment, making them ideal anticancer target candidates, which indicates that USPs inhibitors may be a class of potential anticancer therapeutic agents. However, there are no relevant inhibitors targeting USPs have entered clinical trial so far. In this review, we will summarize the roles and mechanisms of USPs in malignant transformation and progression as well as recent advances of small-molecule inhibitors targeting USPs.

## Introduction

### Ubiquitin proteasome system

The ubiquitin proteasome system (UPS) is the primary system responsible for the degradation of substrates and the regulation of fundamental cellular processes in eukaryotic cells, such as transcriptional activation, stress response, DNA repair, signal transduction, etc. (D'Arcy et al., 2015; Pfoh et al., 2015). UPS consists of a tagging molecule–ubiquitin and a multi-subunit proteolytic complex–the 26S proteasome. Ubiquitin selectively labels the misfolded or unwanted proteins to be degraded and the 26S proteasome functions as a molecular shredder that breaks down these substrate proteins into small molecular peptides for using in other biological processes (D'Arcy et al., 2015; Selvaraju et al., 2015).

Ubiquitin, a small and highly conserved small molecular protein (76 amino acid), is covalently attached to substrates using an isopeptide bond between the terminal Gly-residue of ubiquitin and the 3-amino group of a Lys-residue on the target protein. Ubiquitin contains seven Lys-residues, including Lys6, Lys11, Lys27, Lys29, Lys33, Lys48, and Lys63, all of which can covalently attach to other ubiquitin molecules. The formation of polyubiquitin chains through linkage ultimately determines the destiny of the bound substrates. Generally, proteins labeled with Lys48-linked poly-ubiquitin chains are bound for degradation, whereas proteins tagged with Lys63-linked chains are more typically associated with non-proteasomal roles, including DNA replication, signal transduction and DNA repair, of course, Lys63-ubiquitination can also lead to the substrates to be degraded via autophagy-lysome pathway (ALP) (McKeon et al., 2015). Aside from these instances, other linkage types of polyubiquitin chains are less well-characterized.

The 26S proteasome, an ATP-dependent multi-subunit proteolytic structure, which is located in the nucleus and in the cytoplasm of eukaryotic cells, functions as the molecular shredder for UPS. Based on the diversity of functions, the 26S proteasome can be divided into two discrete sub-structures: a catalytic 20S core particle, which contains the necessary protease active sites, and two19S regulatory particles, which function as a selective and effective facilitator for transfer to the 20S core particle (Selvaraju et al., 2015).

**Figure 1:**
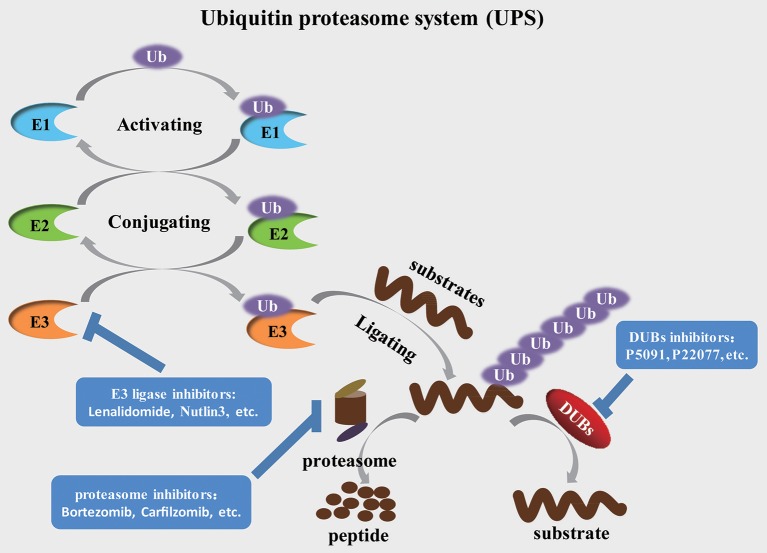
The ubiquitination, degradation and deubiquitination process of protein substrates Ubiquitin is activated by E1 activating enzymes and then, in turn, transferred to E2 conjugating enzymes and E3 ubiquitin ligases. E3 ubiquitin ligases specifically recognize and catalyze the substrate, ultimately leading to the degradation of substrates through the proteasome or stabilization of target proteins through relevant DUBs.

The ubiquitination process is a multi-step cascade reaction that is catalyzed by at least three components—activation, conjugation, and ligation. This process depends on the continuous activity of three discrepant catalyzing enzymes, E1 activating enzymes, E2 conjugating enzymes and E3 ubiquitin ligases. The E1 activating enzymes catalyze the ATP-dependent activation of ubiquitin and form the thioester bond between a Cys-residue in the active site of the E1 activating enzymes and the C-terminus of ubiquitin. Then the activated ubiquitin is transferred to the E2 conjugating enzyme, which forms a thioester bond between a Cys-residue in the active site of the E2 ubiquitin-conjugating enzyme and the C-terminus of ubiquitin. The E3 ubiquitin ligases subsequently recognize and catalyze the ubiquitination of the target protein, ultimately transferring these substrates to the 26S proteasome (Figure 1). In human cells, ~700 E_3_ ubiquitin ligases guarantee the specificity of substrates selection, indicating that E3 ubiquitin ligases determine the specificity and type of ubiquitination identified for the substrates (Komander et al., 2009; Skaar et al., 2014).

### Deubiquitinases (deubiquitinating enzymes)

The process of ubiquitination is highly reversible and subject to dynamic post-translational modifications that are involved in regulating multiple cellular pathways. Deubiquitinases or deubiquitinating enzymes (DUBs) can reverse the effect of E3 ligases by removing ubiquitin from target proteins and are also involved in ubiquitin maturation, recycling and editing (Pfoh et al., 2015; Singh and Singh, 2016; Harrigan et al., 2018). The human genome encodes ~100 DUBs. Based on the mechanism of enzymatic cleavage, DUBs can be divided into two main classes: cysteine proteases and zinc metalloproteases. Based on sequence and domain conservation, DUBs can also be divided into six subfamilies: ubiquitin-specific proteases (USPs), ovarian-tumor proteases (OTUs), Machado–Joseph disease protein domain proteases (MJDs), ubiquitin carboxy-terminal hydrolases (UCHs), monocyte chemotactic protein-induced protein (MCPIP) and JAMM/MPN domain-associated metallopeptidases (JAMMs) (D'Arcy et al., 2015; Pfoh et al., 2015; Kemp, 2016; Harrigan et al., 2018). Among these DUBs, USPs are the most numerous classes with ~60 proteases in humans, with sizes ranging from 50 to 300 kDa (Pfoh et al., 2015). USPs includes an enormous subset of proteins with relevant DUB activity. Their substrates and the regulatory mechanisms, biological functions, and particularly the roles of they play in a variety of clinical diseases, has become increasingly appreciated. The overall evaluation of gene mutations and aberrant expression of USPs in various cancers makes USPs as potential anticancer targets, and there is increasing interest in developing USP-specific inhibitors as candidates for anticancer therapeutic agents (Anupama and Nicholas, 2014). Therefore, we will emphasize the roles and mechanisms of USPs in tumorigenesis and development of multiple cancers, as well as the potentiality and feasibility of USPs inhibition as a novel cancer therapeutic strategy.

### The structure of USPs

Emerging evidence has indicated that most members of the USPs subfamily play significant roles in the progression of various types of cancer (Anupama and Nicholas, 2014). The deubiquitinase activity of USPs is closely associated with their structural features. Thus, it is crucial to understand the structure of USPs.

**Figure 2:**
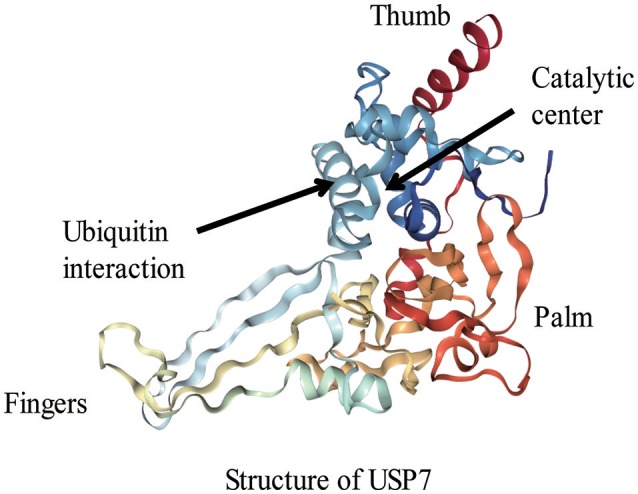
The structure of USP7 (Protein Data Bank (PDB) identifier 1NBF).The fingers, thumb, and palm regions of the USP domain are indicated.

David Komander et al. (Komander et al., 2009) analyzed the domain architectures of USPs and uncovered numerous predicted ubiquitin-binding domains (UBDs) in USPs, including the ubiquitin-associated domain (UBA domain), the ubiquitin-interacting motif (UIM) and the zinc finger ubiquitin-specific protease domain (ZnF-UBP domain). USP domains are composed of three regions, which have been compared to the fingers, thumb and palm of a hand (Komander et al., 2009; Kemp, 2016). Like most of USP domain-containing DUBs, the 26S proteasome-related USP14 also includes a finger subunit that is contact with more than forty percent of the distal ubiquitin and interdicts approach of Lys48 or Lys63. This characteristic allows USP14 to bind to the terminal region of an ubiquitin chain instead of linking to the internal region of the chain. USP14 is specific to Lys-48-linked ubiquitin chains (Hu et al., 2005; Komander et al., 2009). The catalytic center of USPs is located in the interface between the palm and thumb regions, and the fingers grasp the specific position of the ubiquitin chain: as in an ubiquitin dimer, it refers to the conjugated-ubiquitin molecule through the C-terminal Gly-subunit. However, some apo-USP domains, those which cannot bind to the target protein, have a non-productive catalytic structure. When bound to the ubiquitin, the apo-USP domains undergo conformational changes. The changes can be regarded as the result of a dynamic equilibrium between inactive and active conformations, which shifts toward the active conformation. USP7 (HAUSP; Figure 2) is the best example of ubiquitin binding in which the catalytic Cys-residue need to be in range of the His-residue. However, the catalytic triads of USP8 and USP14 are correctly aligned for catalysis in the absence of ubiquitin, and the active site is protected by ubiquitin-binding surface loops (Komander et al., 2009).

## The roles of USPs in cancer

**USP1** is a key protein involved in several significant steps in the process of DNA damage response, including regulating the Fanconi anemia pathway (Nijman et al., 2005; Iraia et al., 2013; D'Arcy et al., 2015), the process of translesion synthesis (Huang et al., 2006; Iraia et al., 2013; Villamil et al., 2013), and the process of differentiation in specific cellular contexts (Williams et al., 2011; Villamil et al., 2013). The Fanconi anemia pathway can prevent the repair of DNA interstrand crosslinks and predisposes patients to diseases, including cancer. Dysfunction of the Fanconi anemia pathway can cause multiple abnormalities that lead to cancer, which are correlated with deregulation of USP1. Translesion synthesis is a process associated with DNA damage tolerance, which permits DNA replication to occur in region of a variety of types of DNA damage caused by exogenous or endogenous factors (Villamil et al., 2013). However, the activation of USP1 requires USP1 cofactor, called USP1-associated factor 1(UAF1; D'Arcy et al., 2015; Kemp, 2016). Studies show that the formation of the USP1/UAF1 complex greatly enhances the catalytic activity of USP1, but USP1 alone has a very low level of activity (Cohn et al., 2007; Villamil et al., 2012). Based on an investigation of tumor microarray expression data in the Oncomine Research Edition Database, the level of USP1 mRNA expression is markedly changed in several malignant tumors, including sarcoma, melanoma, gastric cancer, etc. and USP1 is aberrantly over-expressed in gastric or cervical cancer and in melanoma and sarcoma, as well as in osteosarcoma (Iraia et al., 2013). In view of the frequent over-expression of USP1 in various tumors and the important roles of USP1 in DNA damage response, USP1 inhibition may be a novel anticancer therapy.

**USP2** and its isoforms are involved in the development of several types of cancer. USP2 regulates cell growth or death and involves in the pathogenesis of various diseases, including malignant tumors (Zhu and Gao, 2017). It has been reported that USP2 can regulate and stabilize fatty acid synthase, an aberrantly over-expressed protein in biologically-aggressive prostate cancer cells (Sacco et al., 2010). Deubiquitinating enzyme, USP2a, an isoform of USP2, has been shown to have oncogenic properties in a variety of cancers through regulation of its substrate proteins, including MDM2, MDMX, FASN as well as Aurora A, and USP2a is abnormally over-expressed in multiple cancers, particularly in prostate cancer (Kim et al., 2012). USP2a is known to deubiquitinate and stabilize MDM2 and MDMX, and unlike USP7/HAUSP, USP2a does not interact with p53; over-expression of USP2a was shown to increase the level of MDM2/MDMX protein and to reduce p53 stability (Sacco et al., 2010; D'Arcy et al., 2015). Further studies (Kim et al., 2012; D'Arcy et al., 2015) show that USP2a targets and deubiquitinates cyclin A1, a key cell cycle regulator, leading to the blocking of degradation of cyclin A1 and the accumulation of cyclin A1 as well as the enhancement of cell proliferation. There is evidence that USP2a functions as an oncogene in bladder cancer, the aberrant expression of USP2a results in an increase in proliferation, migration, invasion and resistance to multiple chemotherapeutic agents when compared to control cells (Kim et al., 2012). In addition, USP2 is also involved in tumor progression and associated with poor prognosis in oral squamous cell carcinomas (da Silva et al., 2009; Sacco et al., 2010). Therefore, targeting USP2 may be a therapeutic candidate for oncology.

**USP7**, also known as HAUSP, plays an oncogenic role in the process of cancer development. It has been reported that increased levels of USP7 directly correlates with the development of multiple cancers, such as prostate cancer, multiple myeloma, ovarian cancer, etc. (Tavana and Gu, 2017). The expression of USP7 increases significantly in high-grade prostate cancer biopsies compared with low-grade biopsies. Increased USP7 expression is also observed in multiple myeloma tumors; patients with high USP7 levels showed a poorer overall survival rate when compared to patients with lower USP7 levels (Chauhan et al., 2012). Similarly, USP7 expression is strongly correlated with disease severity and lower overall patient survival in gliomas (Cheng et al., 2013) and epithelial ovarian cancer (Ma and Yu, 2016) as well as in non-small cell lung cancer (Zhao et al., 2015). Over-expression of USP7 promotes cell invasiveness, whereas USP7 knock-down inhibits cell viability in ovarian cancer cells (Ma and Yu, 2016; Tavana and Gu, 2017). USP7 plays specific and direct roles in multiple cancer types and regulates several key proteins, including p53, MDM2, PTEN, and FOXO, which play critical roles in the pathways that consistently cause dysregulation in malignant tumors. USP7 expression closely correlates with cancer stage, tumor size and prognosis, which indicates that USP7 may be a prognostic marker in certain cancers (Tavana and Gu, 2017). Furthermore, small molecules antagonistic to USP7 may function as anticancer agents, targeting certain tumor types.

**USP9X**, also referred to as FAM, is a substrate-specific DUB, which plays pivotal roles in human cancers, both as an oncogene or as a tumor suppressor. USP9X can deubiquitinate and stabilize MCL1, a protein essential for the survival of stem and progenitor cells from multiple lineages, thereby promoting cell survival. Over-expression of USP9X is closely related to an increase in MCL1 protein, whereas aberrant expression of MCL1 stimulates chemoresistance and disease relapse (Opferman and Green, 2010; Schwickart et al., 2010; D'Arcy et al., 2015). Furthermore, multiple myeloma patients with high level of USP9X had a poorer overall survival rate and prognosis compared to patient with lower USP9X levels (Schwickart et al., 2010). USP9X can also regulate and stabilize CEP131, a centriolar satellite protein, ultimately promoting breast carcinogenesis, indicating that USP9X is a significant regulator of centrosome biogenesis and revealing a critical role for the USP9X/CEP131 axis in breast carcinogenesis (Li et al., 2017). In addition, USP9X can also function as a tumor suppressor. USP9X strongly interacts with LATS, a core kinase in the Hippo pathway. Increased USP9X expression significantly up-regulates and stabilizes LATS and leads to a decrease in the transport of YAP/TAZ into the nucleus as well as inhibiting of their target genes. Furthermore, the expression level of USP9X is positively related to LATS expression but negatively associated with the expression of YAP/TAZ in multiple tumor tissues, such as pancreatic cancer and breast cancer, demonstrating that USP9X potentiates LATS kinase in suppressing tumor growth (Toloczko et al., 2017).

**USP10**, a primary cytoplasmic DUB, acts as an oncogene or a tumor suppressor by regulating various protein substrates, including FLT3, p53, AMPK, PTEN, etc. USP10 was identified as a critical DUB for the stabilization of FLT3, whose oncogenic forms are clinically validated targets in acute myeloid leukemia (AML). Treatment with USP10 inhibitors promotes proteasome-mediated FLT3 degradation, leading to a decrease in FLT3 protein levels (Weisberg et al., 2017). USP10 can also directly deubiquitinate and stabilize p53, reversing nuclear export and degradation of p53 induced by MDM2. In the case of DNA damage, USP10 is stabilized and translocates to the nucleus to activate and stabilize p53. The research by Yuan et al. demonstrated that USP10 inhibits cancer cell proliferation in wild-type p53 cells, but promotes tumorigenesis in a mutant p53 background (Yuan et al., 2010). Furthermore, USP10 can also interact with and deubiquitinate PTEN, USP10 inhibition stimulates tumor growth and invasion, but this effect can be abolished by reinserting PTEN (Sun et al., 2018). In addition, AMPK is another classic protein substrate of USP10. Under energy stress conditions, USP10 specifically removes ubiquitination on AMPKα and promotes AMPKα phosphorylation. Meanwhile, AMPKα phosphorylation stimulates USP10 activation by phosphorylating Ser76 of USP10, thus forming a feedforward loop between USP10 and AMPK, ensuring amplification of AMPK activation (Deng et al., 2016). All of these studies showed that USP10 can function as an oncogene or tumor suppressor, so intervention in USP10 may be a candidate anti-cancer strategy.

**USP14/Ubp6**, one of the three distinct DUBs associated with the proteasome, dynamically regulates proteasome activity and removes ubiquitin as well as rescues protein substrates from degradation. USP14 is closely associated with the occurrence and development of multiple malignant tumors, such as breast cancer, lung adenocarcinoma, multiple myeloma and other tumors. Evidence (Zhu et al., 2016) suggests that USP14 is over-expressed in breast cancer tissue compared to adjacent normal tissue; evidence also reveals that over-expression of USP14 in breast cancer patients is tightly correlated to poorer overall survival and prognosis. A study by Wu et al. (Wu et al., 2013) investigated the clinical characteristics and prognostic significance of USP14 in patients with lung adenocarcinoma, as well as its role in cell proliferation in lung cancer. The results showed that the expression of USP14 increases significantly in non-small cell lung cancer (NSCLC) tissue, particularly in lung adenocarcinoma tissues, and over-expression of USP14 promotes tumor cell proliferation. Furthermore, USP14 can also deubiquitinate and stabilize vimentin, a vital protein which involves in epithelial-to-mesenchymal transition(EMT) and significantly promotes cell growth, migration and invasion in human gastric cancer (Zhu et al., 2017). Taken together, these studies suggest that USP14 is involved in the occurrence and progression of multiple malignant tumors.

**Other USP family members:** USP13, the main regulator of ovarian cancer energy metabolism, specifically deubiquitinates and stabilizes oxoglutarate dehydrogenase and ATP citrate lyase, which can catalyze fatty acid synthesis, glutaminolysis and mitochondrial respiration. Treatment with USP13 inhibitors can significantly suppress ovarian tumor progression and enhance the sensitivity of tumor cells to PI3K/AKT inhibitors (Han et al., 2016). A study (Eichhorn et al., 2012) identified that USP15 functions as a crucial component of the transforming growth factor β (TGF-β) pathway, a cancerogenic factor in advanced cancer. USP15 can deubiquitinate type I TGF-β receptor (TβR-I) and enhance TGF-β activity; and over-expression of USP15 is closely related to TGF-β activation as well as a poor prognosis for glioblastoma patients. The research by Cheryl et al. (Cheryl et al., 2013) examined and evaluated the clinical significance of USP17 in NSCLC for the first time, demonstrating that USP17 is highly expressed in both adenocarcinoma and squamous NSCLC tissue, and the expression level of USP17 may be closely related to distant NSCLC metastasis and recurrence-free survival. Beyond that, other USPs family members are also more or less involved in the occurrence and development of malignant tumors, so USPs may be the potential anticancer target.

## Small molecular inhibitors targeting USPs as anticancer agents

As mentioned previously, USPs play important roles in various types of cancer and are involved in many different biological processes, including cell cycle control, regulation of histones, DNA damage response and regulation of transcription factors. Targeting USPs may be an effective anticancer therapeutic strategy, indicating that USPs inhibitors may be a class of potential novel therapeutic agents for various types of cancers. However, more extensive research needs to done to explore the specific mechanisms and targets of DUBs, with the objective of studying and designing small-molecule USP inhibitors. There are a large number of small-molecule DUB inhibitors that have been reported, ranging from broad-spectrum inhibitors to specific inhibitors targeting individual DUB enzymes. As shown in Table 1, some inhibitors targeting USPs have been reported. In the following sections, we will primarily introduce the inhibitors of USP7, USP10 and USP14. Of course, among these inhibitors, individual inhibitor can also target several DUBs, including no-USP subfamily.

**Table 1:** Reported inhibitors targeting USPs.

**USPs**	**USPs inhibitors**	Structure of USPs inhibitor^*^	**References**
USP1	Pimozide ML323^#^ SJB2-043 SJB3-019A GW7647	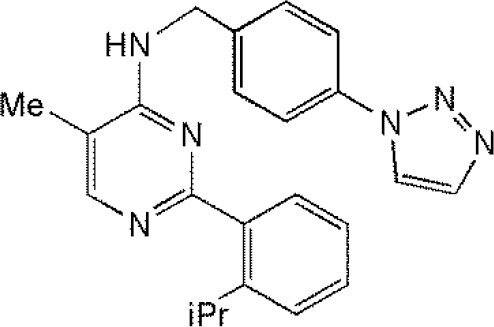	(Liang et al., 2014; Wei et al., 2015) (McClurg and Robson, 2015)
USP2	F6 (NSC 632839) AM146 RA-9 RA-14 ML364^#^	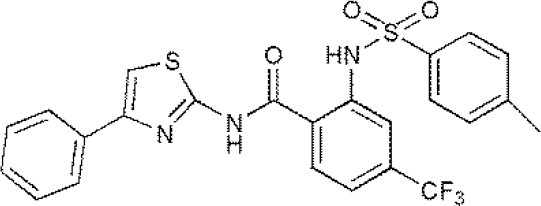	(Singh and Singh, 2016) (D'Arcy et al., 2015) (Davis et al., 2016)
USP4	Vialinin A^#^	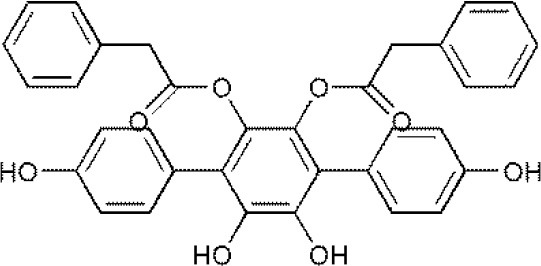	(Okada et al., 2013)
USP5	WP1130 EOAI3402143^#^ Vialinin A	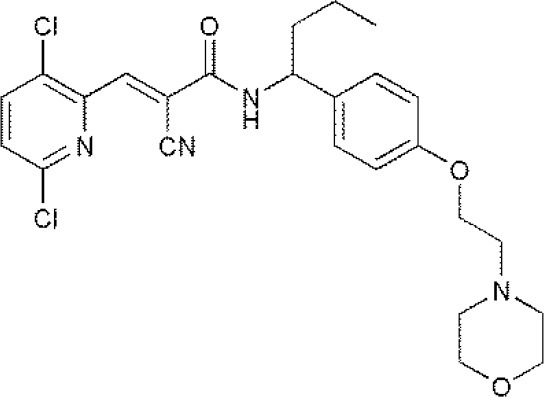	(Okada et al., 2013; Wei et al., 2015)
USP7	HBX 19818 HBX 28258 P5091^#^ P22077 Cpd14 P022077 HBX41108 P0050429 WO2013030218	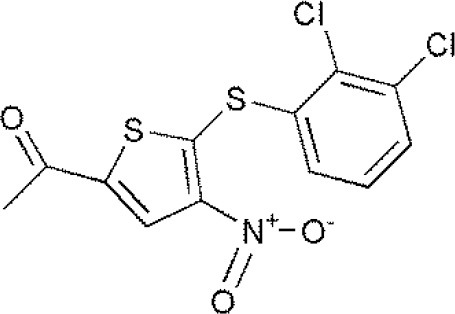	(Sacco et al., 2010) (D'Arcy et al., 2015; Wei et al., 2015)
USP8	HBX 90397 RA-9^#^ RA-14 HBX41108 AM146 Ethyloxyimino-9H-indeno[1,2-b]pyrazine-2,3-dicarbonitrile	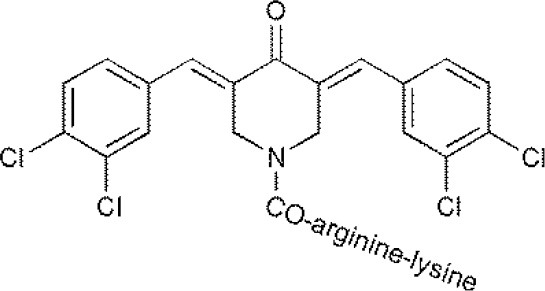	(D'Arcy et al., 2015) (Wei et al., 2015)
USP9X	WP1130^#^ EOAI3402143 (G9)	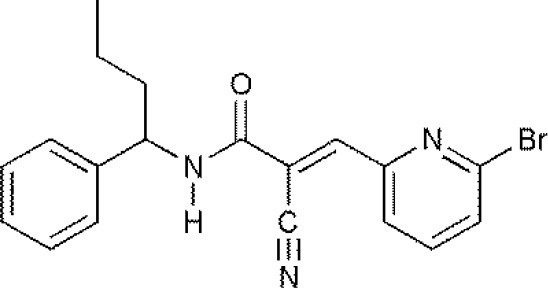	(Wei et al., 2015)
USP10	P22077 HBX-19818^#^ Spautin-1	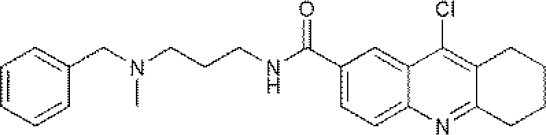	(Liu et al., 2011) (McClurg and Robson, 2015; Weisberg et al., 2017)
USP11	Mitoxantrone^#^	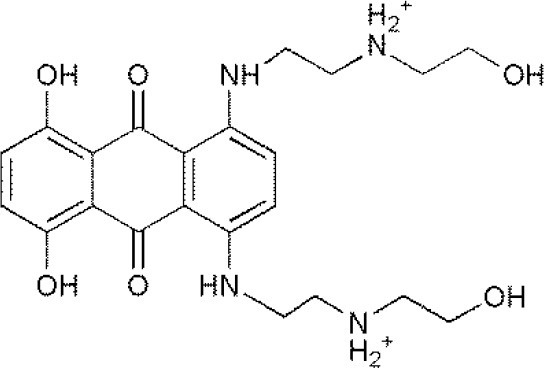	(Wei et al., 2015)
USP13	Spautin1^#^	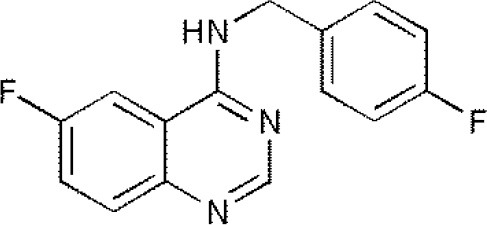	(Kemp, 2016)
USP14	b-AP15 VLX1570 IU1^#^ Auranofin WP1130 AC17	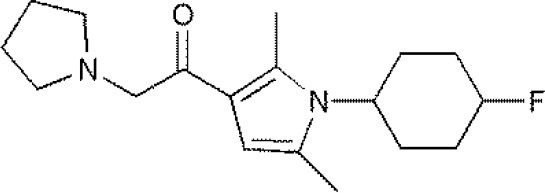	(D'Arcy et al., 2015) (Qiu et al., 2017) (Wei et al., 2015)
USP46	Pimozide^#^	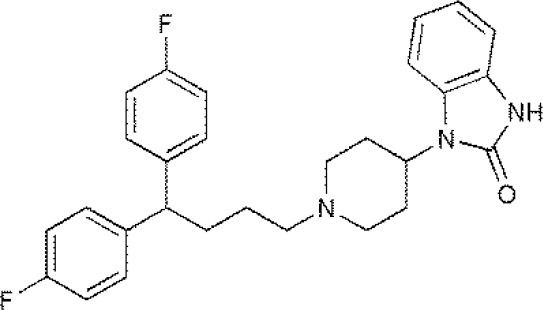	(McClurg and Robson, 2015)
USP47	Cpd 14 P22077^#^ P5091	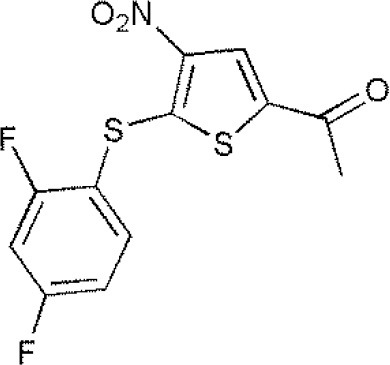	(D'Arcy et al., 2015)

* *The column of “Structure of USPs inhibitor” is corresponding to the structure of inhibitors marked with “^#^”*.

### Inhibitors targeting USP7

USP7 (HAUSP) has received tremendous attention from researchers due to the key roles played by USP7 in cellular processes, and studies of inhibitors targeting USP7 have become a hot topic. As time goes by, HBX19818, P5091, as well as their analogs, including P045204, P22077, and HBX41108, have been confirmed as inhibitors of USP7. HBX41108 is a potent specific inhibitor targeting USP7, and kinetic analysis revealed that HBX41108 inhibits USP7 by interacting with the enzyme-substrate complex rather than by directly competing with substrate binding (Colland et al., 2009). Carmody and Ruaidhri described in WO 2012056048 inhibition of USP7 with HBX19818 can lead to an increase in ubiquitination and destabilization of NF-κB, ultimately limit the inflammatory response in the treatment of acute or chronic inflammation (Carmody, 2012). The mechanism of action of HBX19818 is to bind with USP7 and weaken the deubiquitinating activity of USP7, leading to the occurrence of apoptosis mediated by p53, in HCT116 colon cancer cells (Reverdy et al., 2012; Lim et al., 2016). Recent research showed that P22077 could induce cell apoptosis and cell proliferation inhibition mediated by p53 in the orthotopic neuroblastoma mouse models of IMR-32, SH-SY5Y, and NGP; since USP7 is overexpressed in neuroblastoma patients, P22077 may be an anti-neuroblastoma agent (Fan et al., 2013; Lim et al., 2016). As disclosed in US 20160090351, P5091 displays a preference for USPs (Hedstrom et al., 2016) and another study (Pal et al., 2014) demonstrated that P5091 is a novel small-molecule inhibitor targeting USP7 and USP47, as well as functions as an active anticancer agent in various tumor models, including MM.1S multiple myeloma cells and HCT-116 colon cancer cells (Chauhan et al., 2012; Pal et al., 2014). Treatment with p-5091 can stabilize p53 protein levels and inhibit cell growth as well as induce the occurrence of apoptosis in multiple myeloma cells resistant to traditional chemotherapy agents.

### Inhibitors targeting USP10

At present, there are few studies regarding USP10 inhibitors. The reported inhibitors of USP10 primarily include P22077, HBX19818, and Spautin-1. As mentioned previously, P22077 and HBX19818 were both originally reported as irreversible inhibitors of USP7. However, a recent study (Weisberg et al., 2017) revealed that P22077 and HBX19818 may also inhibit the deubiquitinase activity of USP10 and induce the proliferation inhibition of mutant-FLT3 (FLT3-ITD)-positive cancer cells. A study by Liu et al. (Liu et al., 2011) has demonstrated that Spautin-1 is a potent small-molecule inhibitor of USP10 and USP13, and treatment with Spautin-1 inhibits the deubiquitinase activity of USP10 and USP13, resulting in an increasing ubiquitination and accelerating degradation of Beclin1 in Vps34 complexes, ultimately inhibiting autophagy. In WO 2014145512, it is demonsrated that the potent small molecule inhibitors of autophagy are useful in treatment of cancers and acute pancreatitis (Yuan et al., 2014). In addtion, a recent study showed that spautin-1 can also trigger immunogenic cancer cell death *in vivo* and *in vitro* by causing mitochondrial oxidative injury rather than autophagy inhibition, also demonstrated that spautin-1 may stimulate an apoptotic pathway that results in immunogenic cancer cell death, in TFAM- and AGER-dependent fashion (Yang et al., 2018).

### Inhibitors targeting USP14

**B-AP15** is a unique class of proteasome inhibitors that target the 19S RP-associated deubiquitinases, including USP14 and UCHL5 (no-USP subfamily). Treatment with b-AP15 can induce accumulation of polyubiquitin in cells, leading to endoplasmic reticulum stress (ER stress) and oxidative stress, ultimately resulting in apoptosis. Beyond this, b-AP15 can also effectively inhibit the dissemination of acute myeloid leukemia (AML) mouse model of C1498 leukemia, and inhibit the carcinogenesis process in multiple solid tumor mouse models, including Lewis lung carcinomas (LLCs) xenografts, HCT-116 colon carcinoma xenografts overexpressing BCL2, 4T1 breast carcinomas xenografts, etc. (D'Arcy et al., 2011; Pal et al., 2014; Tian et al., 2014). Recent research revealed that b-AP15 can block the degradation of protein substrates, indicating that USP14 inhibition weakens the function of the proteasome (D'Arcy et al., 2015). In addition, the research by Ding et al. showed that b-AP15 can also produce its antitumor therapeutic effects to hepatocellular carcinoma cells by enhancing the endoplasmic reticulum (ER) stress and the unfolded protein response (UPR), as well as inhibiting Wnt/β-catenin and Notch1 signaling pathways (Ding et al., 2018).

**IU1** As disclosed in US 20130045992, IU1 can enhance the activity of proteasomes and prevent proteinopathies through inhibition of the function of USP14 and promotion of degradation of the protein substrates (Finley et al., 2013). The mechanism of action of IU1 is different from bortezomib, which inhibits the activity of the entire proteasome, whereas IU1 binds specifically and inhibits USP14 (Lim et al., 2016). IU1, or other similar inhibitors that can target and inhibit USP14, increasing proteasomal-mediated degradation, may function as therapeutic agents in some diseases like Alzheimer's disease (D'Arcy et al., 2015).

**WP1130** As described in US 20160090351, small molecule inhibitor WP1130 is a potent DUBs inhibitor (Hedstrom et al., 2016) and can target and inhibit several deubiquitinases, including USP5, USP9X, USP14, and UCHL5 (no-USP subfamily), all of which can regulate stability of the protein substrates and the function of the proteasome (D'Arcy et al., 2015; Lim et al., 2016). Treatment with WP1130 can induce the accumulation of poly-ubiquitin of target protein rapidly, leading to the occurrence of apoptosis. However, the mechanism of action of WP1130 is different from b-AP15; WP1130 cannot induce the occurrence of oxidative stress. The inhibition of UCHL5 and USP14 deubiquitinase activity by WP1130 is expected to block the function of the proteasome in tumor tissue cells, but this still needs to be tested (D'Arcy et al., 2015). WP1130 can also inhibit the deubiquitinase activity of USP9X, and treatment with WP1130 can promote apoptosis by decreasing the level of MCL-1 and increasing the sensitivity of cancer cells to traditional chemotherapy (Lim et al., 2016).

## Concluding remarks

Over the past decade, studies regarding DUBs have made great progress. Accumulating evidences have revealed that a large number of DUBs play significant roles in the occurrence and development of various malignant tumors. USPs are vital and highly specialized class of DUBs with emerging therapeutic potentialities for cancers and targeting USPs has emerged as an appealing novel anticancer therapy. The continued and accelerated development of small molecule inhibitors against USPs could increase the probability of success in the treatment of various malignant cancers and other fatal diseases, but there remain many challenges. Great efforts need be made to comprehensively understand the roles, substrates and regulation mechanisms of USPs in various diseases, and to clarify their emerging roles in carcinogenesis and the clinical application of their inhibitors. To sum up, many USPs are involved in regulation of various pathways relevant to cancer, targeting USPs may be a novel anticancer therapeutic therapy.

## Author contributions

BY and HZ conceived and designed the conception of review article, as well as amended the paper. TY conducted the paper. FY, MY, JC, and QH collected the related research articles and reviews.

### Conflict of interest statement

The authors declare that the research was conducted in the absence of any commercial or financial relationships that could be construed as a potential conflict of interest.

## Abbreviations

DUBs: deubiquitinases or deubiquitinating enzymes
UPS: ubiquitin proteasome system
USPs: ubiquitin-specific proteases
